# Looking Upstream: Promoting Health Equity in Philadelphia through Novel Partnership Strategies

**DOI:** 10.1089/pop.2021.0217

**Published:** 2021-12-03

**Authors:** Adrea E. Cope, David B. Nash, Sandra E. Brooks, David Platt

**Affiliations:** ^1^ Philadelphia Collaborative for Health Equity, Thomas Jefferson University, Philadelphia, Pennsylvania, USA.; ^2^ College of Population Health, Thomas Jefferson University, Philadelphia, Pennsylvania, USA.; ^3^ US Clinical Development and Medical Affairs, Novartis Pharmaceuticals Corporation, East Hanover, New Jersey, USA.

**Keywords:** community health, health disparities, cardiovascular disease, social determinants of health

## Introduction

The coronavirus disease 2019 (COVID-19) pandemic has been cataclysmic, revealing the troubling depth of health inequities in the United States. Disproportionately higher rates of COVID-19 infection, hospitalizations, and deaths among Black Americans were documented in multiple cities throughout the United States. Upstream causes of these disparities are related to decades of discriminatory policies resulting in inequities among housing quality, economic opportunities, education, criminal justice, and health care.^1,2^ As a result, Black Americans are more likely to live in impoverished neighborhoods – environments associated with adverse outcomes related to COVID-19 infection. These communities were severely disadvantaged by significant barriers to testing in the early phases of the pandemic, by employment in sectors that required in-person interaction or suffered significant layoffs, and by living environments that were not as amenable to social distancing.^3,4^ The conditions in which people live, work, and play – or social determinants of health (SDOH) – deeply fostered the devastating impact COVID-19 had on these communities.

The pandemic has led to a new sense of urgency to address SDOH. Urban academic medical centers provide health services to a significant percentage of patients from impoverished communities, and therapeutic innovations from pharmaceutical corporations will only have a desired impact if they are able to be implemented across at-risk populations equitably. Acknowledging and understanding upstream SDOH is imperative to truly achieving better health outcomes.

From an entrepreneurial perspective, there is emerging interest from digital health companies to develop technologies that either monitor health, measure health statistics, or connect vulnerable populations to resources. Cityblock Health and Unite Us are companies that have undergone rapid growth aiming to connect vulnerable populations with services that promote health and ultimately lower high-cost utilization.^5,6^ Other examples include Google's Healthcare Natural Language API, which translates SDOH information into action-based reports for physicians, and rideshare companies such as Uber and Lyft that have partnered with health care institutions to overcome transportation barriers for patients, providing free or reduced cost rides.^7^

The pandemic led to a rapid acceleration of telehealth that fueled major mergers, most notably that of Teladoc and Livongo, two of the largest publicly traded virtual care companies.^8^ This merger created a health technology giant, marking the beginning of a rapidly expanding future for digital health. In 2020, more health care companies went public than in the past 5 years combined.^9^ Recognizing that aging populations represent a sizable group that could potentially benefit from coordination of care, prominent primary care organizations such as Oak Street Health and Accolade were among those with high valuations in the initial public offering market, marking a major shift in the economic valuation of clinical care provider entities that treat primarily Medicare patients.

These trends indicate the potential for major changes in health care delivery, as the future largely points to a robust landscape of digital health technology and a growing understanding of the role of social determinants in determining health outcomes. As we focus on the profound lack of equity in health outcomes for racial and ethnic minorities in the United States today, interventions to address these issues must also be profound. This is where we predict a new paradigm – a unique partnership between academic medicine and the pharmaceutical industry to leverage resources addressing these issues in a timely manner.

## Call to Action

Historically, relationships between pharmaceutical industries and academic medicine have been fraught with challenges. An example is the inclusion of the Sunshine Act in the 2010 Affordable Care Act that served as a deterrent to collaboration given the need to report detailed information about payments and other “transfers of value” from manufacturers of drugs, medical devices, and biologics to physicians and teaching hospitals.^10^ However, recognition of the toll the pandemic has taken on vulnerable populations has served as a catalyst for action. It was with this realization that an academic medical center in Philadelphia, Pennsylvania, Thomas Jefferson University, and pharmaceutical manufacturer, Novartis Pharmaceuticals Corporation (Novartis), came together with a joint mission of promoting health equity to reduce cardiovascular disease morbidity and mortality.

Thomas Jefferson University established the Philadelphia Collaborative for Health Equity (P-CHE) in 2017, a major vehicle to convene communities with local and regional stakeholders, prioritize community health needs, and implement strategies to address those needs.^11^ P-CHE initiatives take a grassroots approach to build trust and co-develop robust infrastructure designed to enhance the lives and well-being of those in the community.^11^ A recent example is the establishment of the Frazier Family Coalition for Stroke Education and Prevention – an initiative designed to address the 35-times higher rate of stroke in the North Philadelphia community. The Coalition will build community capacity and connect individuals to vital resources, including cardiovascular risk reduction programs and services.

Likewise, Novartis has supported several population health programs. For example, the Novartis Foundation, an independent foundation that aims to promote health and well-being among underserved populations, has led the Better Hearts Better Cities initiative. This initiative partnered with government authorities in low- and middle-income countries to address hypertension – the leading risk factor for cardiovascular disease and a major cause of death worldwide.^12^ Using the comprehensive CARDIO approach described in the Urban Population Health Toolkit,^13^ the initiative saw tremendous results, with preliminary findings indicating tripled blood pressure control in Dakar, Senegal within just 2 years of project implementation.^12^ Similarly, in Sao Paulo, Brazil, preliminary data showed that blood pressure control tripled after only 1.5 years of implementation, reaching a better control rate than in some European countries.^12^ Novartis aims to leverage these learnings and best practices to improve cardiovascular population health in the United States.

In 2020, as a response to worsening health outcomes resulting from the COVID-19 pandemic, the Novartis US Foundation announced a $25 million commitment to develop partnerships and fund community programs designed to approach health equity. This commitment posed a novel question: How can the pharmaceutical industry and academic medicine leverage unique resources to promote health equity within an urban environment in a way that is inclusive and representative of the communities in which they seek to serve?

Born out of a desire to address this question, Jefferson and Novartis designed Closing the Gap *–* an initiative to promote health equity and improve cardiovascular disease outcomes in target high-risk zip codes across Philadelphia. Cardiovascular disease remains the leading cause of death for both men and women in the United States, and Philadelphia is no exception – in 2019, cardiovascular disease accounted for approximately 24% of total deaths in the city.^14^ Philadelphia is also the poorest large city in the United States, with 25% of the population living at or below the federal poverty level.^15^ Major risk factors for cardiovascular disease – hypertension, diabetes, and high cholesterol – are highest among non-Hispanic Black populations and in areas with the highest rates of poverty.

Closing the Gap will invest directly in 5 zip codes in the North and South regions of Philadelphia, expanding and strengthening existing infrastructure and connecting individuals to the care they need. Initiatives will address health at the social determinant level, ensuring local participation by providing operational and financial support to community organizations that already may be providing services to community members in a setting that is familiar and comfortable. Additionally, a robust screening and community health worker program will ensure that individuals are assessed for cardiovascular disease risk factors and connected with programs designed to reduce their risk while also making certain they receive the resources needed to optimize health – whether that is in the form of a nutrition meal service, transportation and travel assistance, or connection to a specialty care provider. Closing the Gap also will aim to address policy and structural issues and concerns through community capacity building and advocacy.

Closing the Gap is an example of the type of initiative that can emerge when partners are focused on addressing root causes of health disparities. As we begin to move forward in a post-pandemic world, we must not forget the grounding reality we faced. No single entity is equipped to tackle the multifaceted issues that drive SDOH, and innovative partnerships similar to the one we have described will propel us in the right direction.

## Author Note

This editorial represents the personal views of the authors and does not necessarily represent the views of their employers.
